# 2-Deoxystreptamine Conjugates by Truncation–Derivatization of Neomycin

**DOI:** 10.3390/ph3030679

**Published:** 2010-03-15

**Authors:** M. Waqar Aslam, Leandro C. Tabares, Alessio Andreoni, Gerard W. Canters, Floris P.J.T. Rutjes, Floris L. van Delft

**Affiliations:** 1 Radboud University Nijmegen, IMM Organic Chemistry, Heyendaalseweg 135, 6525 AJ, Nijmegen, the Netherlands; 2 Leiden Institute of Chemistry, Leiden University, Einsteinweg 55, 2300 RA, Leiden, the Netherlands

**Keywords:** aminoglycosides, morpholine, aminopyridine, aminoquinoline, hemocyanin, biosensor, fluorescence

## Abstract

A small library of truncated neomycin-conjugates is prepared by consecutive removal of 2,6-diaminoglucose rings, oxidation-reductive amination of ribose, oxidation-conjugation of aminopyridine/aminoquinoline and finally dimerization. The dimeric conjugates were evaluated for antibacterial activity with a unique hemocyanin-based biosensor. Based on the outcome of these results, a second-generation set of monomeric conjugates was prepared and found to display significant antibacterial activity, in particular with respect to kanamycin-resistant *E. coli.*

## Introduction

Aminoglycosides, a group of naturally occurring compounds obtained from actinomycetes of the genus *Streptomyces* or *Micromonospora* [1] are in clinical use as antibiotics as a result of their broad antimicrobial spectrum and rapid bactericidal effects [2]. Aminoglycosides bind to 16S ribosomal RNA at the tRNA acceptor A-site (aminoacyl site), and affect the ability of the ribosome to decode mRNA correctly during protein synthesis [3,4,5]. Unfortunately, toxic side effects and growing bacterial resistance [6,7] have narrowed the significance of aminoglycosides as antibiotics. The most common mechanism of resistance is the enzymatic modification of one or more functional groups of the aminoglycoside drug by bacterial enzymes [8,9,10]. Due to these limitations, aminoglycosides are the focus of attention of research groups around the world and numerous structural analogues of the aminoglycosides have been synthesized over the years [11,12,13]. The main objective of the synthetic modifications of the aminoglycosides is to circumvent the bacterial resistance without loss in binding affinity of these drugs. In the majority of studies, naturally occurring aminoglycosides are modified by regioselective diversifications of the appropriate functional groups while keeping the whole structure intact [14,15,16,17,18,19,20,21]. However, it is clear that structures with a high resemblance to the natural compounds are most likely to undergo modification by bacterial resistance enzymes. Therefore, unlike this strategy we intended to utilize a minimal core element for the development of new structural analogues. Because bacterial enzymes have evolved to modify the structures of naturally occurring aminoglycosides, stripping off the targeted alcohol and amino functions evades the problem of bacterial resistance. On the other hand, such a strategy will concomitantly also reduce antibacterial activity because the same heteroatoms are responsible for RNA binding. Therefore, in order to restore affinity, lost by functional group removal, we envisaged conjugation of such a truncated aminoglycoside with a non-aminoglycoside type RNA ligand. Such a strategy has earlier proven successful for conjugation of native aminoglycosides to acridines [22,23], nucleobases [24], nucleotides [25], peptides [26,27], and other antibiotics [28]. Also, diversification of neamine as a structural motif for the synthesis of RNA ligands has been explored by several research groups [29,30,31,32,33]. However, neamine still contains the diaminosugar ring I of aminoglycosides, and is therefore a substrate for several resistance enzymes. Dimeric aminoglycoside have also divulged an improved RNA binding than individual aminoglycosides [34,35,36,37,38,39,40,41,42,43], therefore we made the dimers of our conjugates with conformationally adaptable linkers to further enhance binding affinity. These compounds were then tested for antibacterial activity against *E. coli* with a fluorescence-based assay.

## Results and Discussion

### Synthesis of 5-O-Morpholino-2-Deoxystreptamine

**Scheme 1:**
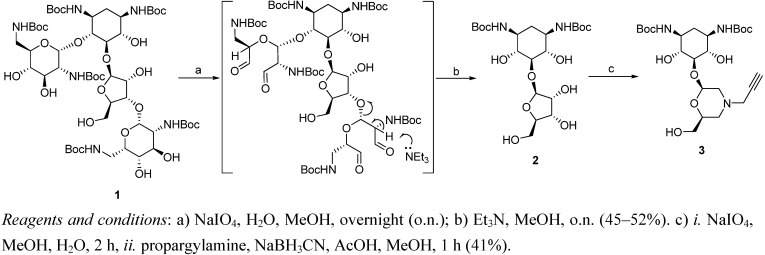
Synthesis of acetylene-substituted morpholino-2-deoxystreptamine conjugate.

Our strategy is based on the fundamental observation that the key structural feature of (nearly) all aminoglycosides is not a carbohydrate but a diaminocyclohexitol ring termed 2-deoxystreptamine [44]. It was hypothesized that 2-deoxystreptamine is a crucial scaffold to build aminoglycoside libraries and that the all-equatorial substitution pattern is highly favorable to position other pharmacophores in the proper orientation. Although a large number of synthetic routes to 2-deoxystreptamine have been developed over the years [44], including contributions from our own lab [45,46,47,48], we realized that the most straightforward and cheapest route to 2-deoxystreptamine commences from natural neomycin. Apart from that, we surmised that *partial* degradation of neomycin would leave the ribofuranoside as a suitable substituent at the 5-position of 2-deoxystreptamine, as in structure **2** (Scheme 1). Thus, *N*-Boc-protected neomycin **1** [49] was reacted with 12 equivalents of sodium metaperiodate resulting in the oxidative cleavage of vicinal diols on both 2,6-diaminosugars. The intermediate tetraaldehyde was not purified due to its instability, but upon base treatment underwent smooth β-elimination under the influence of an amine base, to give 5-*O*-ribosyl-2-deoxystreptamine **2**. Use of different bases like *n*-butyl amine, ammonium hydroxide and triethylamine gave the same results in terms of yield and reaction time. Importantly, **2** was purified by direct recrystallization from dichloromethane leading to highly pure product **2** in a 48% average yield over the two steps. The resulting pseudodisaccharide **2** was once again subjected to sodium metaperiodate cleavage of the diol to give a dialdehyde, this time followed by reductive amination with propargylamine, to afford morpholine **3** substituted with an acetylene moiety. Unfortunately, the formation of open chain diamine side products could not be fully avoided, thus significantly suppressing the yield.

### Synthesis and Conjugation of Aminopyridines and Aminoquinolines

**Scheme 2:**
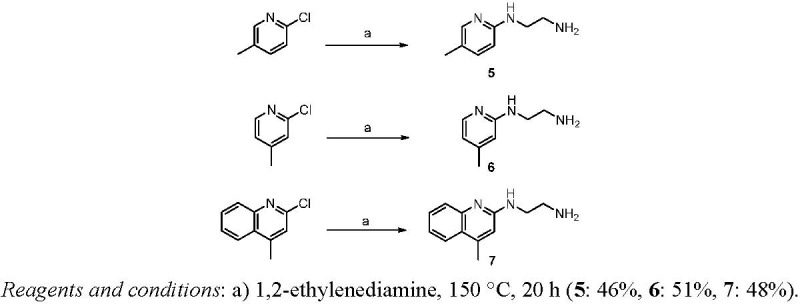
Synthesis of substituted aminopyridines and aminoquinolines.

As aminoglycoside antibiotics function by selective recognition and binding to a specific RNA sequence, the removal of two of the aminoglycopyranosides is detrimental for RNA affinity. Therefore, we opted to restore the binding affinity by conjugation with other RNA ligands. To this end, we surmised that aminopyridines and aminoquinolines [50] could nicely serve our purpose because these molecules are of low molecular weight and contain no aliphatic amine functionality, but nevertheless have been reported to bind with *E. coli* A-site RNA in micromolar range. We selected the two tightest binders from the series of aminopyridines, e.g., 2-(2-aminoethylamino)-4-methylpyridine and 2-(2-aminoethylamino)-5-methylpyridine, and the best aminoquinoline ligand 2-(2-aminoethylamino)-4-methylquinoline (Scheme 2). In order to be able to conjugate the arylamine ligands to our morpholine compound, we designed a route involving reductive amination via the primary alcohol of **3**. Therefore, we first prepared derivatives of the arylamines by treating the commercially available chloropyridines and a chloroquinoline with 1,2-ethylenediamine at 150 °C for 18 hours as shown in the Scheme 2, to afford the desired 2-aminoethyl modified arylamines **5**–**7** in reasonable yield. 

Having the primary amines at hand, the next step involved the diversification of morpholine **3** by conjugation with the aminoethyl derivatives **5**–**7**. For this purpose the primary hydroxyl of the compound **3** appeared most appropriate since it is most accessible and leaves the chiral secondary alcohols of 2-deoxystreptamine intact. Since reductive amination is such a robust conjugation technology, we aimed to selectively oxidize the primary alcohol of **3**. Because it is known that Swern oxidations can be selectively executed at *O*-TES protected primary alcohols [51], we treated compound **3** with TESOTf and triethylamine in dichloromethane to protect all hydroxyls with the TES group. Subsequently, the triply TES-protected derivative was subjected to Swern oxidation conditions (oxalyl chloride, DMSO, -78 °C→0 °C), in order to selectively oxidize the primary hydroxyl. However, an inseparable mixture of compounds was obtained (Table 1, entry 1).

**Scheme 3:**
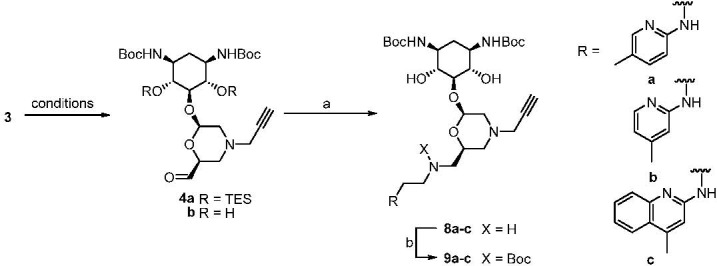
Conjugation of morpholineto aminopyridines and aminoquinolines.

Because Swern oxidation of TES ethers was not successful, we opted for a selective oxidation of the primary alcohol without protective groups. However, attempts to oxidize the primary hydroxyl with TCCA/TEMPO [52] (entry 2) or IBX [53] (entry 3) gave no or less than 10% conversion, respectively. Finally, we succeeded (entry 4) in oxidizing the primary hydroxyl group with Dess-Martin periodinane [54] to give selectively the desired aldehyde **4b**. However, also in this case the reaction did not go to completion, reaching a maximum of about 60% conversion. Further attempts to optimize the reaction conditions, e.g., variation in temperature, solvents or stoichiometry of the oxidant did not improve the outcome. 

**Table 1:** Conditions for selective oxidation of primary alcohal.

Entry	Conditions	Product	Yield
1	i) TESOTf, Et_3_N, CH_2_Cl_2_, r.t., 3 h ii) (COCl)_2_, DMSO, CH_2_Cl_2_, -78 °C→0°C	**4a**	mixture
2	TCCA, TEMPO, CH_3_CN, r.t., 16 h	**4b**	no reaction
3	IBX, DMF, r.t.→80 °C, 16 h	**4b**	<10% conversion
4	DMP, DMF, r.t., 16 h	**4b**	60% conversion

*Reagents and conditions*: a) **5**/**6**/**7**, NaBH_3_CN, AcOH, MeOH, 3 h. b) Boc_2_O, DMAP, CH_2_Cl_2, _4 h (**9a**: 34%, **9b**: 37%, **9c**: 32%, for two steps).

The resulting aldehyde **4b** was subjected to the next reaction without purification on account of its instability. We therefore proceeded to couple crude aldehyde **4b** with aminopyridine and aminoquinoline derivatives **5–7** (Scheme 3). Therefore, compounds **5–7** were condensed with aldehyde **4b** employing standard reductive amination conditions, e.g., NaBH_3_CN and AcOH in methanol. The relatively low yields of the resulting compounds **8a-c** can be ascribed to incomplete oxidation of the compound **3** in the proceeding step, apart from the fact that accompanying dialkylation of amines was observed during the reductive amination. However, in this case the desired conjugates **9a**-**c** could be readily purified by silica gel column chromatography. Both for this purpose and for follow-up chemistry, it was found most convenient to convert the resulting secondary amino functions of the initial products **8a**-**c** into Boc-protected carbamates under the influence of Boc_2_O and DMAP in CH_2_Cl_2_. Thus, heteroconjugates **9a**-**c** of truncated neomycin with non-aminoglycoside type RNA ligands were successfully prepared.

### Dimerization and Deprotection of the Conjugates

Obviously, the obtained arylamine-morpholine-2-deoxystreptamine conjugates contain a functional handle at the morpholine ring in the form of the propargyl moiety. Based on the popular copper(I)-catalyzed azide acetylene cycloaddition (CuAAC) [55,56], it is clear that the propargyl moiety provides an excellent handle for the preparation of further conjugates. Therefore, we next utilized the copper-catalyzed (3+2) cycloaddition chemistry to make immers with a range of mono- and bisazido-functionalized linkers. This approach provided maximum synthetic flexibility and proved effective for the synthesis of various monomers and immers from the building blocks **9a**-**c**. Thus, the synthetic heteroconjugates were treated with a monoazide (benzyl azide, 1 equiv.) or bisazides **A**-**E** (0.5 equiv.) in a mixture of water and acetonitrile in the presence of copper-wire [57] to make the respective monomers **9a**-**cE** and dimers **9a**-**cA** to **9a**-**cD** in reasonable to good yields (Table 2). Finally, acidic deprotection of the Boc protective groups was performed in a 1:2 mixture of TFA and DCM to afford the respective monomers and the dimers in quantitative yields, giving a final set of 15 arylamine-aminoglycoside conjugates (3 monomers, 12 dimers). For biological evaluation, all of the compounds were purified by reversed phase HPLC, giving the pure conjugates in 5–20 mg quantitities.

**Scheme 4:**
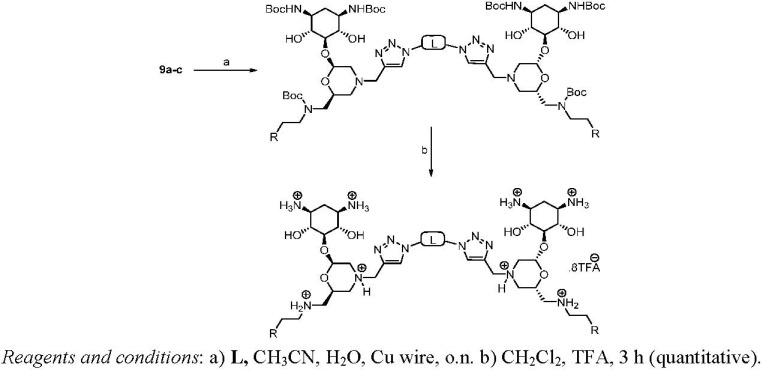
Dimerization and deprotection of the conjugates.

**Table 2:** Linkers used for synthesis of dimers 9aA-9cE.

**Entry**	**Linker (L)**	**Starting**	**Product**	**Yield**
		**material**		
1	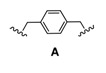	**9a**	**9aA**	63%
2		**9b**	**9bA**	73%
3		**9c**	**9cA**	60%
4	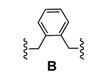	**9a**	**9aB**	57%
5		**9b**	**9bB**	65%
6		**9c**	**9cB**	58%
7	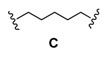	**9a**	**9aC**	61%
8		**9b**	**9bC**	66%
9		**9c**	**9cC**	69%
10	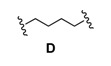	**9a**	**9aD**	60%
11		**9b**	**9bD**	65%
12		**9c**	**9cD**	66%
13	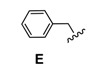	**9a**	**9aE**	87%
14		**9b**	**9bE**	92%
15		**9c**	**9cE**	90%

### Evaluation of Antibacterial Activity

**Figure 1:**
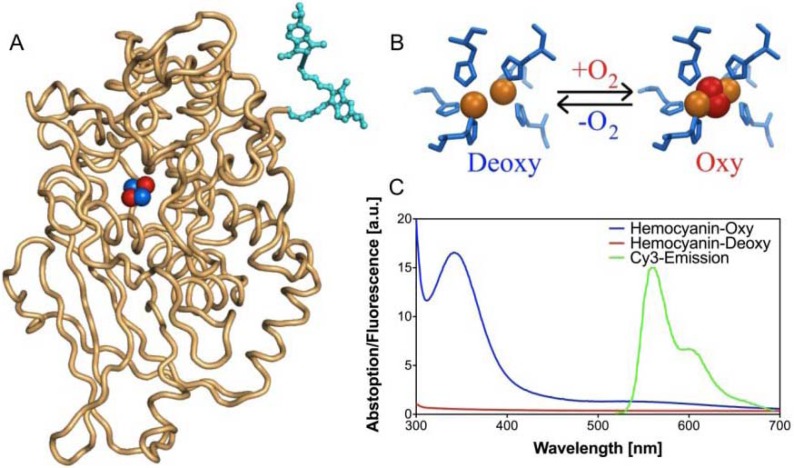
(A) Representation of Cy3-labeled hemocyanin. The Cy3 dye (cyan) is showed attached to the end terminus of a monomer hemocyanin. (B) Detail of hemocyanin active site showing the active site histidines (blue), copper ions (orange) and the binding oxygen molecule (red). (C) The absorption spectra of the deoxygenated (red) and oxygenated (blue) forms of hemocyanin together with the emission spectrum of Cy3 (green).

Having prepared the desired conjugates, we were interested to investigate the antibacterial activity of these compounds. Determination of antibacterial activity is normally expressed in MIC (minimal inhibitory concentration) by growing bacteria in medium containing increasing amounts of the presumed antibiotic. Such an assay typically requires substantial amounts of compounds (30–70 mg), clearly exceeding the amount of compound that we had prepared. With small amounts of substrate, antibacterial activity can also be determined with Kirby-Bauer disc test, but that provides only a qualitative measure of activity. Alternatively, a plethora of RNA-binding assays have been developed [58,59,60,61,62,63,64,65,66,67,68,69], but a strong drawback of such assays is that they do not include cell-wall penetration or resistance mechanisms, obviously key in determining the overall activity of an antibiotic [70]. An alternative approach, recently reported by some of us [71], determines MICs on whole bacteria in a simple and rapid assay and moreover requires only minimal amounts (< 2 mg) of substrate. The technique relies on a fluorescence-based cell viability assay and involves a hemocyanin-based oxygen biosensor to monitor oxygen consumption of bacteria and subsequently bacterial cell growth (Figure 1).

Deoxygenated hemocyanin has no absorption in the visible spectrum but upon oxygenation a strong band centered at 340 nm and a weak band around 570 nm appear (Figure 1B). If a fluorescent label is attached to the N-terminus of hemocyanin, this change in absorption can be translated into a change of fluorescence intensity of an attached label (Cy3, Cyanine 3 fluorescent dye) through a Förster resonance energy transfer (FRET) mechanism (Figure 1A) [72]. The efficiency of this energy transfer depends on the distance and orientation between the donor and the acceptor and also on the overlap integral between the donor emission and acceptor absorption spectra (Figure 1C). In this way, when the protein is in its deoxygenated state (Cy3-Deoxy-Hemocyanin), all the energy absorbed by the label is emitted normally as fluorescence. Upon oxygenation of hemocyanin (Cy3-Oxy-Hemocyanin), energy is transferred from Cy3 by radiationless decay to the copper center of hemocyanin, resulting in a decrease in the fluorescence intensity. Thus, when a bacterial culture grows aerobically, oxygen in the medium is gradually consumed. This results in a drop of the oxygen concentration, eventually resulting in an anaerobic medium. When hemocyanin-based oxygen biosensor is present, this oxygen depletion results in an increase of the fluorescence intensity emitted by the Cy3 label attached to protein [71]. Tests were carried out as described [71] with an initial cell concentration of 10^5^ cfu/mL.

**Figure 2:**
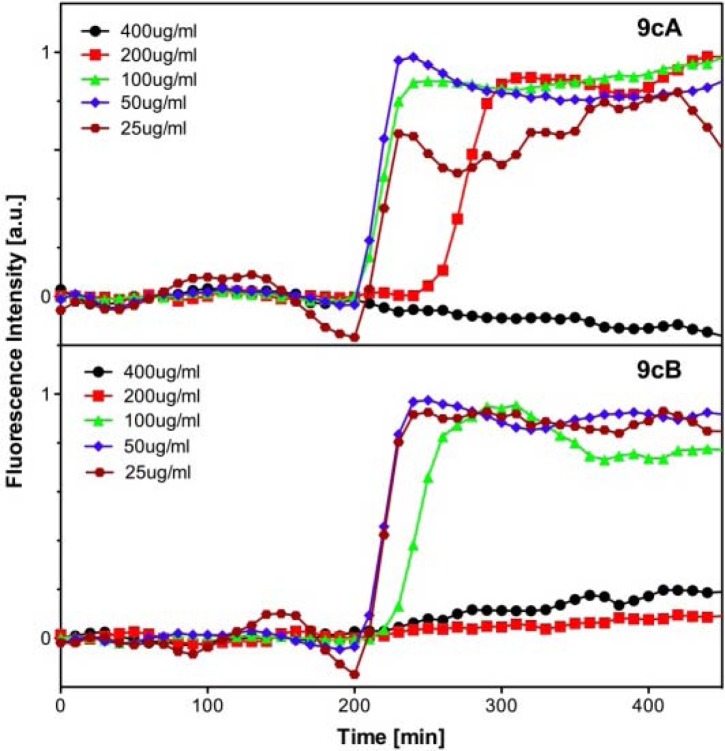
Fluorescence intensity time traces at 570 nm (excitation 550 nm) of cultures of *E. coli* containing Cy3-labeled hemocyanin as a function of antibiotic concentration. Antibiotic concentrations of **9cA** and **9cB** are stated.

We determined the MICs of our heteroconjugates with the fluorescence-based cell viability assay against several bacterial strains (*E. coli, E. coli kan.^r^* e.g., kanamycin-resistant *E. coli*) using kanamycin as a positive control. As can be seen from Figure 2, using compound **9cA** and **9cB** as an example, the MIC is determined by performing the bacterial growth in medium containing increasing amounts of our compounds. The MIC values of our synthetic analogues can be determined from Figure 2 and are depicted in Table 3.

**Table 3:** MIC values (µg/ mL) as determind by the fluorescence-based cell growth assay.

Entry	bacterial strain	MIC (μg/ mL)						
		kanamycin	s.m.	linker A	linker B	linker C	linker D	linker E
1	*E. coli*	10	**9a**	200–400	200–400	400–800	400–800	400–800
2	-do-	10	**9b**	400–800	400–800	400–800	400–800	400–800
3	-do-	10	**9c**	200–400	100–200	200–400	400–800	200–400

### Second Generation Monomeric Ligands

Unfortunately, our set of dimers of heteroconjugates exhibited lower antibacterial activity than that of natural kanamycin when tested against *E. coli*. In fact, only compound **9cB** demonstrated reasonable antibacterial activity. Moreover, and in contrast to expectation, it is clear that the dimers do not show improved activity compared to the monomeric substrates **9a**-**cE**.

**Scheme 5:**
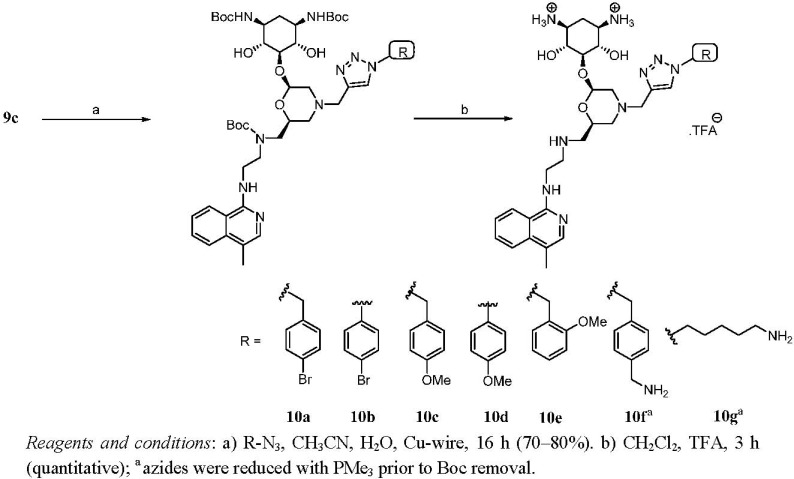
Synthesis and deprotection of monomeric ligands.

The lack of improved activity is in contrast to findings by other groups [34,35,36,37,38,39,40,41,42,43], which may be explained in our case by the relatively short linkers employed. It is not excluded that a short linker is precluding cooperative binding of the other end of the molecule to a second binding pocket. Whatever the reason, the fact that the MIC value of the monomeric compound **9cE** was comparable to that of different dimers stimulated us to explore whether antibacterial activity of the more simple, monomeric heteroconjugates could be further raised by modification of the azide partner for cycloaddition to the acetylene. Thus, we selected a range of aromatic and aliphatic azides and coupled these to acetylene derivatives **9a**-**c** with CuAAC to obtain a set of monomeric compounds **10a-g** with different substituents (Scheme 5). Thus, compounds **10a–e** were synthesized by simply treating **9c** with different monoazides in a mixture of water and acetonitrile in the presence of copper wire. For the synthesis of amino-terminated compounds **10f** and **10g,** acetylene **9c** was treated with an excess of 1,4-bis(azidomethyl)benzene or 1,5-diazidopentane, to give the respective monosubstituted triazole adducts, followed by Staudinger reduction of the unreacted azide group at the other chain terminus under the action of PMe_3_. Again, all compounds **10a**-**g** were purified to homogeneity by reversed-phased HPLC before evaluation of antibacterial activity. Next, we tested the antibacterial activity of this set of compounds employing the same fluorescence-based cell viability assay and the results are summarized in Table 3. As evident from MIC values, *para*-bromo substitution of the aromatic ring enhanced the antibacterial activity of compounds **10a** and **10b**. The rest of the monomeric compounds, with a methoxy- or methylamino-substituent on the aromatic ring **10c-10f**, or with the aliphatic chain **10g** showed a reduced antibacterial activity compared to that of unsubstituted parent compound **9cE.** Finally we tested the MIC of the most active compounds against the kanamycin resistant strains of *E. coli* (Table 4, entry 2). We were happy to find that compounds **10a** and **10b** have distinct antibacterial activity against *E. coli.* More importantly, compound **10b** displayed an improved activity against the kanamycin-resistant *E. coli* strain revealing that omission of the diaminoglycosides indeed fulfills our desired aim to reduce susceptibility to resistance mechanisms. The search for further synthetic analogues of aminoglycosides with such improved traits is currently ongoing in our laboratory.

**Table 4:** MIC values (μg/mL) as determined by fluorescence-based cell growth assay.

Entry	bacterial strains	MIC (μg/mL)									
		kanamycin	10a	10b	10c	10d	10e	10f	10g	9cB	9cE
1	*E. coli*	10	100-200	100-200	400-800	400-800	400-800	400-800	400-800	-	-
2	*E. coli kan^r^*	400–800	400-800	200-400	>800	-	-	-	-	>800	>800

## Experimental Section

### General

TLC analyses are performed on silica gel-coated plates (Merck 60 F254) with the indicated solvent mixture. Compounds were detected with ammonium molybdate or potassium permanganate staining, or UV light. Flash column chromatography was carried out using ACROS silica gel (0.035–0.070 mm, ca 6 nm pore diameter). Solvents were distilled from appropriate drying agents prior to use. Unless stated otherwise, all chemicals were purchased and used as such. IR spectra were recorded on an ATI Mattson Genesis Series FTIR spectrometer; absorption reported in cm^-1^. NMR spectra were recorded on a Bruker DMX 300 (75 MHz), and a Varian 400 (400 MHz) spectrometer. ^1^H-NMR and ^13^C-NMR spectra are reported in ppm units on the δ scale. Coupling constants are reported as *J*-values in Hz. Semipreparative RP-HPLC was performed using a C_18 _column (10 × 250 mm, 5 µm) with HPLC grade MilliQ containing 0.1% TFA (eluant A) and acetonitrile containing 0.1% TFA (eluant B) with a gradient of 5 → 50% of B in 30 min. Compounds were detected by UV absorption at 215 and 254 nm.

#### Hexa-N-(tert-butoxycarbonyl) neomycin B *(**1**) [49]*

To a solution of neomycin B (2 g, 2.2 mmol) in a mixture of H_2_O/1,4-dioxane (30 mL, 1:1 v/v) was added Na_2_CO_3 _and di-*tert*-butyl dicarbonate. The reaction mixture was stirred at room temperature for 6 h. Dioxane was removed by evaporation and the residue was partitioned between EtOAc (100 mL) and H_2_O (100 mL). The aqueous layer was extracted with EtOAc (2 ×50 mL) and the combined organic layers werewashed with brine (100 mL), dried (Na_2_SO_4_) and concentrated *in vacuo*. Purification by column chromatography (MeOH/CH_2_Cl_2_, 1/9) afforded **1** (2.2 g, 82%) as an amorphous white solid. *R*_F _0.49 (MeOH/CH_2_Cl_2_, 1/9). HRMS (ESI) *m/z* calcd. for C_53_H_94_N_6_O_25_ (M+Na)^+^: 1237.6166, found: 1237.6141.

#### 5-O-(β-d-ribofuranosyl)-2-deoxystreptamine *(**2**)*

To a solution of **1** (1 g, 0.82 mmol) in 25 mL of MeOH, was added 25 mL of a H_2_O solution of NaIO_4_ (2.12 g, 9.8 mmol). The reaction mixture was stirred at room temperature for 16 h. Methanol was removed by evaporation and the residue was partitioned between EtOAc (100 mL) and H_2_O (50 mL). The aqueous layer was extracted with EtOAc (2 × 50 mL) and the combined organic layers werewashed with brine (100 mL), dried (Na_2_SO_4_) and concentrated *in vacuo*. The residue was dissolved in MeOH (30 mL), and Et_3_N (2 mL) was added. The reaction mixture was stirred at room temperature for 16 h and then concentrated in *vacuo*. Purification by crystallization from CH_2_Cl_2 _(100 mL) afforded compound **2** (212 mg, 52%) as a white amorphous solid. *R*_F _0.12 (MeOH/CH_2_Cl_2_, 1/9); ^1^H-NMR (MeOD, 400 MHz) δ 5.21 (s, 1H), 4.32 (dd, *J* =4.5, 7.7 Hz, 1H), 3.99 (d, *J* =4.5 Hz, 1H), 3.90 (dt, *J* =2.6, 7.7 Hz, 1H), 3.78 (dd, *J* =2.3, 12.1 Hz, 1H), 3.60 (dd, *J* =2.6, 12.1 Hz, 1H), 3.40–3.15 (m, 10H), 2.02 (dt, *J* =4.2, 7.9 Hz, 1H), 1.41 (s, 18H), 1.21 (m, 1H); ^13^C-NMR (MeOD, 75 MHz): δ 156.3, 107.8, 83.8, 82.3, 78.2, 74.7, 72.7, 68.6, 59.7, 50.6, 33.8,26.8; HRMS (ESI) *m/z* calcd. for C_21_H_38_N_2_O_11_ (M+Na)^+^: 517.2373, found: 517.2375.

#### 5-O-(N-propyn-1-yl -morpholino)-2-deoxystreptamine *(**3**)*

To a solution of **2** (830 mg, 1.68 mmol) in 20 mL of MeOH, was added 20 mL of a H_2_O solution of NaIO_4_ (431 mg, 2.016 mmol). The reaction mixture was stirred at room temperature for 2 h. The methanol was removed by evaporation and the residue was partitioned between EtOAc (100 mL) and H_2_O (50 mL). The aqueous layer was extracted with EtOAc (2 ×50 mL) and the combined organic layers werewashed with brine (100 mL), dried (Na_2_SO_4_) and concentrated *in vacuo*. The residue was dissolved in 15 mL of MeOH, and NaBH_3_CN (422 mg, 6.72 mmol) was added. To the resulting mixture was added drop-wise a solution of propargylamine (138 μL, 2.52 mmol) and AcOH (403 μL, 6.72 mmol) in MeOH (1 mL). The reaction mixture was stirred at room temperature for 2 h. The reaction was then quenched with 0.5 mL of Et_3_N and concentrated *in vacuo*. The residue was taken up in EtOAc (100 mL), washed with saturated NaHCO_3_ (2 × 50 mL) and brine (50 mL). The organic layer was dried (Na_2_SO_4_), filtered and concentrated *in vacuo*.Purification by column chromatography (MeOH/CH_2_Cl_2_, 1/9) afforded **1** (358 mg, 41%) as an amorphous white solid. *R*_F _0.34 (MeOH/CH_2_Cl_2_, 1/9); ^1^H-NMR (MeOD, 400 MHz) δ 4.85 (dd, *J* = 2.3, 8.6 Hz, 1H), 3.70 (m, 1H), 3.57 (dd, *J* = 1.7, 5.1Hz, 2H), 3.42–3.19 (m, 11H), 3.01 (d, *J* = 10.5 Hz, 1H), 2.70 (d, *J* = 11.1 Hz, 1H), 2.65 (t, *J* = 2.3 Hz, 1H), 2.13 (m, 2H), 2.02 (dt, *J* = 3.9, 13.0 Hz, 1H), 1.41 (s, 18H), 1.21 (dd, *J* = 12.5 Hz, 1H). ^13^C-NMR (MeOD, 75 MHz): δ 156.2, 99.7, 85.2, 78.1, 76.7, 74.1, 73.5, 72.5, 61.9, 54.1, 51.3, 50.6, 50.4, 45.1, 33.6, 26.8; HRMS (ESI) *m/z* calcd. for C_24_H_41_N_3_O_9_ (M+H)^+^: 516.2921, found: 516.2925.

#### 2-(2-Aminoethylamino)-4-methylpyridine *(**5**)*

A solution of 2-chloro-4-methylpyridine (0.876 mL, 10 mmol) in 1,2-diaminoethane (6 mL) was heated at 150°C for 18 h. The volatiles were removed in *vacuo*. Purification by column chromatography (10% MeOH/CH_2_Cl_2 _to 20% MeOH/CH_2_Cl_2_) afforded **5** (700 mg, 46%) as a yellow oil; ^1^H-NMR (MeOD, 300 MHz) δ 7.76 (d, *J* = 4.8 Hz, 1H), 6.36 (m, 2H), 3.31 (t, *J* = 6.2 Hz, 2H), 2.79 (t, *J* = 6.2 Hz, 2H), 2.17 (s, 3H); ^13^C-NMR (MeOD, 75 MHz): δ 156.5, 145.0, 136.2, 121.1, 106.6, 40.1, 39.9, 15.5.HRMS (ESI) *m/z* calcd. for C_8_H_13_N_3_ (M+H)^+^: 152.1187, found: 152.1199.

#### 2-(2-Aminoethylamino)-5-methylpyridine *(**6**)*

A solution of 2-chloro-5-methylpyridine (0.434 mL, 5 mmol) in 1,2-diaminoethane (3 mL) was heated at 150°C for 18 h. The volatiles were removed in *vacuo*. Purification by column chromatography (10% MeOH/CH_2_Cl_2 _to 20% MeOH/CH_2_Cl_2_) afforded **6** (386 mg, 51%) as a yellow oil; ^1^H-NMR (MeOD, 300 MHz) δ 7.72 (m, 1H), 7.27 (dd, *J* = 2.5, 8.5 Hz, 1H), 6.46 (d, *J* = 8.5 Hz, 1H), 3.30 (t, *J* = 6.2 Hz, 2H), 2.79 (t, *J* = 6.2 Hz, 2H), 2.13 (s, 3H). ^13^C-NMR (MeOD, 75 MHz): δ 156.6, 145.3, 138.0, 120.3, 107.7, 43.5, 40.1, 15.5; HRMS (ESI) *m/z* calcd. For C_8_H_13_N_3_ (M+H)^+^: 152.1187, found: 152.1199.

#### 2-(2-Aminoethylamino)-4-methylquinoline *(**7**)*

A solution of 2-chloro-4-methylquinoline (1.7 g, 10 mmol) in 1,2-diaminoethane (6 mL) was heated at 150°C for 18 h. The volatiles were removed in *vacuo*. Purification by column chromatography (10% MeOH/CH_2_Cl_2 _to 20% MeOH/CH_2_Cl_2_) afforded **7** (963 mg, 48%) as a yellow oil; ^1^H-NMR (MeOD, 300 MHz) δ 7.72 (dd, *J* = 1.2, 8.1 Hz, 1H), 7.59 (dq, *J* = 0.5, 1.2, 8.4 Hz, 1H), 7.45 (ddd, *J* = 1.4, 6.9, 8.4 Hz, 1H), 7.17 (ddd, *J* = 1.2, 6.9, 8.1 Hz, 1H), 6.59 (d, *J* = 1.0 Hz, 1H), 3.50 (t, *J* = 6.2 Hz, 2H), 2.86 (t, *J* = 6.2 Hz, 2H), 2.48 (d, *J* = 1.0 Hz, 3H). ^13^C-NMR (MeOD, 75 MHz): δ 156.9, 147.0, 144.2, 128.4, 124.5, 123.0, 122.8, 120.9, 111.9, 42.6, 40.3, 16.9; HRMS (ESI) *m/z* calcd. for C_12_H_15_N_3_ (M+H)^+^: 202.1344, found: 202.1335.

#### 5-O-(N-propyn-1-yl-2-(methylamino-N-ethylamino-N -4-methylpyridin-2-yl)-morpholino)-2-deoxystreptamine ***8a***

To a solution of **3** (65 mg, 0.126 mmol) in DMF (1 mL), was added Dess-Martin periodinane (64 mg, 0.151mmol). The reaction mixture was stirred overnight at room temperature. The reaction was quenched with a saturated solution of NaHCO_3 _(20 mL) and extracted with EtOAc (3 ×25 mL). The combined organic layers were washed with H_2_O (2 × 25 mL) and brine (25 mL), dried (Na_2_SO_4_) and concentrated *in vacuo*. The residue was dissolved in 2 mL of MeOH, and NaBH_3_CN (12 mg, 0.189 mmol) was added. To the reaction mixture was added a solution of **5** (57 mg, 0.378 mmol) and AcOH (11 μL, 0.189 mmol) in MeOH (0.5 mL). The reaction mixture was stirred at room temperature for 4 h. The reaction was quenched with 0.1 mL of Et_3_N and concentrated in *vacuo*. The residue was taken up in EtOAc (50 mL), washed with saturated NaHCO_3_ (2 × 25 mL) and brine (25 mL). The organic layer was dried (Na_2_SO_4_), filtered and concentrated *in vacuo*. Purification by column chromatography (MeOH/CH_2_Cl_2_, 1/9) afforded **8a** (32 mg, 39%) as a white solid. *R*_F _0.22 (MeOH/CH_2_Cl_2_, 1/9); ^1^H- NMR (MeOD, 400 MHz) δ 7.89 (d, *J* = 5.4 Hz, 1H), 6.52 (d, *J* = 5.4 Hz, 1H), 6.47 (s, 1H), 5.03 (d, *J* = 7.5 Hz, 1H), 3.96 (m, 1H), 3.57 (q, *J* = 5.6, 10.3 Hz, 2H), 3.41–3.15 (m, 12H), 2.94 (d, *J* = 10.3 Hz, 1H), 2.70 (m, 2H), 2.29 (dd, *J* = 7.7, 11.0 Hz, 1H), 2.22 (s, 3H), 1.99 (m. 1H), 1.41 (d, *J* = 8.4 Hz, 18H), 1.24 (m, 1H); ^13^C-NMR (MeOD, 75 MHz): δ 156.3, 156.2, 149.1, 144.9, 126.9, 114.3, 109.2, 99.5, 83.2, 78.2, 76.4, 74.8, 73.7, 72.8, 69.1, 54.0, 51.5, 48.7, 45.0, 38.2, 33.8, 26.8, 19.2; HRMS (ESI) *m/z* calcd. for C_32_H_52_N_6_O_8_ (M+H)^+^: 649.3924, found: 649.3923.

#### 5-O-(N-propyn-1-yl-2-(methylamino-N-ethylamino-N -5-methylpyridin-2-yl)-morpholino)-2-deoxystreptamine ***8b***

To a solution of **3** (80 mg, 0.155 mmol) in DMF (1 mL), was added Dess-Martin periodinane (79 mg, 0.186 mmol). The reaction mixture was stirred overnight at room temperature. The reaction was quenched with a saturated solution of NaHCO_3 _(20 mL) and extracted with EtOAc (3 × 25 mL). The combined organic layers were washed with H_2_O (2 × 25 mL) and brine (25 mL), dried (Na_2_SO_4_) and concentrated *in vacuo*. The residue was dissolved in 3 mL of MeOH, and NaBH_3_CN (14 mg, 0.232 mmol) was added. To the resulting mixture was added a solution of **6** (70 mg, 0.465 mmol) and AcOH (14 μL, 0.232 mmol) in MeOH (0.5 mL). The reaction mixture was stirred at room temperature for 4 h. The reaction was quenched with 0.1 mL of Et_3_N and concentrated in *vacuo*. The residue was taken up in EtOAc (50 mL), washed with saturated NaHCO_3_ (2 × 25 mL) and brine (25 mL). The organic layer was dried (Na_2_SO_4_), filtered and concentrated *in vacuo*. Purification by column chromatography (MeOH/CH_2_Cl_2_, 1/9) afforded **8b** (41 mg, 41%) as a white solid. *R*_F _0.22 (MeOH/CH_2_Cl_2_, 1/9); ^1^H- NMR (MeOD, 400 MHz) δ 7.74 (s, 1Η),7.28 (dd, *J* = 2.3, 8.5 Hz, 1H), 6.5 (d, *J* = 8.5 Hz, 1H), 4.85 (dd, *J* = 2.3, 8.6 Hz, 1H), 3.80 (m, 1H), 3.42–3.19 (m, 11H), 2.99 (d, *J* = 10.3 Hz, 1H), 2.18–2.00 (m, 5H), 1.40 (d, *J* = 7.2 Hz, 18H), 1.26 (m, 1H); ^13^C-NMR (MeOD, 75 MHz): δ 156.6, 156.3, 156.1, 145.1, 138.2, 120.5, 108.1, 99.8, 85.2, 78.2, 76.6, 76.2, 74.2, 73.5, 72.6, 71.7, 54.1, 52.5, 50.5, 50.0, 45.0, 39.9, 33.8, 26.8, 15.4; HRMS (ESI) *m/z* calcd. for C_32_H_52_N_6_O_8_ (M+H)^+^: 649.3924, found: 649.3923.

#### 5-O-(N-propyn-1-yl-2-(methylamino-N-ethylamino-N-4-methylquinolin-2-yl)-morpholino)-2-deoxystreptamine ***8c***

To a solution of **3** (100 mg, 0.194 mmol) in DMF (1 mL), was added Dess-Martin periodinane (99 mg, 0.232 mmol). The reaction mixture was stirred overnight at room temperature. The reaction was quenched with saturated solution of NaHCO_3 _(20 mL) and extracted with EtOAc (3 × 25 mL). The combined organic layers were washed with H_2_O (2 × 25 mL) and brine (25 mL), dried (Na_2_SO_4_) and concentrated *in vacuo*. The residue was dissolved in 4 mL of MeOH, and NaBH_3_CN (18 mg, 0.291 mmol) was added. To the resulting mixture was added a solution of **7** (116 mg, 0.582 mmol) and AcOH (17.5 μL, 0.291 mmol) in MeOH (0.5 mL). The reaction mixture was stirred at room temperature for 4 h. The reaction was quenched with 0.1 mL of Et_3_N and concentrated in *vacuo*. The residue was taken up in EtOAc (50 mL), washed with saturated NaHCO_3_ (2 × 25 mL) and brine (25 mL). The organic layer was dried (Na_2_SO_4_), filtered and concentrated *in vacuo*. Purification by column chromatography (MeOH/CH_2_Cl_2_, 1/9) afforded **8c** (52 mg, 38%) as a white solid. *R*_F _0.21 (MeOH/CH_2_Cl_2_, 1/9); ^1^H-NMR (MeOD, 400 MHz) δ 7.84 (d, *J* = 8.1 Hz, 1H), 7.62 (d, *J* = 3.4 Hz, 1H), 7.30 (q, *J* = 4.1, 8.1 Hz, 1H), 6.72 (d, *J* = 1.0 Hz, 1H), 5.06 (d, *J* = 6.3 Hz, 1H), 3.97 (m, 1H), 3.75 (t, *J* = 5.0 Hz, 2H), 3.44–3.18 (m, 13H), 2.95 (d, *J* = 11.0 Hz, 1H), 2.72–2.65 (m, 2H), 2.57 (d, *J* = 1.0 Hz, 3H), 2.30 (dd, *J* = 7.7, 11.0 Hz, 1H), 2.17 (dd, *J* = 9.3, 11.1 Hz, 1H), 2.03 (dt, *J* = 3.8, 12.5 Hz, 1H), 1.42 (d, *J* = 9.1 Hz, 18H), 1.32 (m, 1H); ^13^C-NMR (MeOD, 75 MHz): δ 156.7, 145.9, 145.2, 129.4, 123.7, 123.3, 123.1, 122.0, 112.0, 99.6, 83.5, 78.2, 76.4, 74.7, 73.7, 72.8, 69.2, 54.0, 51.6, 49.1, 48.5, 45.0, 38.4, 26.9, 17.0; HRMS (ESI) *m/z* calcd. for C_36_H_54_N_6_O_8_ (M+H)^+^: 699.4081, found: 699.4062.

### General procedure for Boc-protection of ***9a-c***

To a solution of **8a-c** (0.15 mmol) in CH_2_Cl_2 _(2 mL) was added di-*tert*-butyl dicarbonate (0.18 mmol, 1.2 equiv.) and DMAP (5 mg). The reaction mixture was stirred at room temperature for 5 h. The volatiles were removed in *vacuo*. Purification by column chromatography (MeOH/CH_2_Cl_2_, 1/9) gave the desired product.

*5-O-(N-propyn-1-yl-2-(methylamino-N-(Tert-Butoxycarbonyl)ethylamino-N -4-methylpyridin-2-yl)-morpholino)-2-deoxystreptamine*** 9a**: Yield 87%, white solid. R_F _0.43 (MeOH/CH_2_Cl_2_, 1/9); HRMS (ESI) m/z calcd. for C_37_H_60_N_6_O_10_ (M+H)^+^: 749.4449, found: 749.4434.

*5-O-(N-propyn-1-yl-2-(methylamino-N-(Tert-Butoxycarbonyl)ethylamino-N -5-methylpyridin-2-yl)-orpholino)-2-deoxystreptamine*** 9b**: Yield 90%, white solid. *R*_F _0.43 (MeOH/CH_2_Cl_2_, 1/9); HRMS (ESI) *m/z* calcd. for C_37_H_60_N_6_O_10_ (M+H)^+^: 749.4449, found: 749.4434.

*5-O-(N-propyn-1-yl-2-(methylamino-N-(Tert-Butoxycarbonyl)ethylamino-N -4-methylquinolin-2-yl)-morpholino)-2-deoxystreptamine*** 9c**: Yield 84%, white solid. *R*_F _0.46 (MeOH/CH_2_Cl_2_, 1/9); HRMS (ESI) *m/z* calcd. for C_41_H_62_N_6_O_10_ (M+H)^+^: 799.4605, found: 799.4587.

### 3.3. General procedure for the synthesis of bis-azides ***A-D***

To a solution of a commercially obtained dibromide (2.5 mmol) in 10 mL of DMF was added NaN_3_ (6.0 mmol). The reaction mixture was stirred at 60 °C for 5 h. The reaction was then quenched with H_2_O and extracted with Et_2_O. The organic layers were washed with H_2_O and brine, dried (Na_2_SO_4_) and concentrated in *vacuo*. Purification by column chromatography (EtOAc/heptane, 1/20) gave the pure product.

*1,4-bis(Azidomethyl)benzene* (**A**): Yield 86%, oil. *R*_F _0.34 (EtOAc/heptane, 1/8); ^1^H-NMR (CDCl_3_, 300 MHz) δ 7.34 (s, 4H), 4.35 (s, 4H); ^13^C-NMR (CDCl_3_, 75 MHz): δ 135.0, 128.1, 53.9.

*1,2-bis(Azidomethyl)benzene* (**B**): Yield 82%, oil. *R*_F _0.36 (EtOAc/heptane, 1/8); ^1^H-NMR (CDCl_3_, 300 MHz) δ 7.38 (s, 4H), 4.43 (s, 4H); ^13^C-NMR (CDCl_3_, 75 MHz): δ 133.4, 129.6, 128.5, 51.7.

*1,5-Diazidopentane* (**C**)**:** Yield 91%, liquid. *R*_F _0.49 (EtOAc/heptane, 1/8); ^1^H-NMR (CDCl_3_, 300 MHz) δ3.25 (t, *J* = 6.7 Hz, 4H), 1.60 (m, 4H), 1.42 (m, 2H); ^13^C-NMR (CDCl_3_, 75 MHz) δ 50.7, 27.9, 23.4.

*1,4-Diazidobutane* (**D**)**:** Yield 86%, liquid. *R*_F _0.51 (EtOAc/heptane, 1/8); ^1^H-NMR (CDCl_3_, 300 MHz) δ 3.27(t, *J* = 5.7 Hz, 4H), 1.61 (m, 4H); ^13^C-NMR (CDCl_3_, 75 MHz) δ 50.4, 25.6.

#### 1-Bromo-4-(azidomethyl)bromobenzene

To a solution of 1-bromo-4-(bromomethyl)benzene (200 mg, 0.80 mmol) in 1 mL of DMF was added NaN_3_ (52 mg, 0.80 mmol). The reaction mixture was stirred at 60 °Cfor 5 h. The reaction was then quenched with H_2_O and extracted with Et_2_O. The organic layers were washed with H_2_O and brine, dried (Na_2_SO_4_)and concentrated in *vacuo*. Purification by column chromatography (EtOAc/heptane, 1/20) gave 1-bromo-4-(azidomethyl)benzene (156 mg, 92%) as a colorless liquid. *R*_F _0.51 (EtOAc/heptane, 1/8); ^1^H-NMR (CDCl_3_, 400 MHz) δ 7.47 (d, *J* = 8.4 Hz, 2H), 7.15 (d, *J* = 8.4 Hz, 2H), 4.24 (s, 2H); ^13^C-NMR (CDCl_3_, 75 MHz) δ 133.9, 131.5, 129.3, 121.8, 53.6.

#### 1-Azido-4-bromobenzene

To a solution of NaN_3_ (1.5 g, 24.3mmol) in a mixture of H_2_O/CH_2_Cl_2_ (7.5 mL, 1:1 v/v) at 0 °C, was added Tf_2_O (2.01 mL, 12.15mmol). The reaction mixture was stirred at room temperature for 2 h. After quenching with aqueous NaHCO_3_, the layers were separated and the aqueous layer was extracted with CH_2_Cl_2_ (5.75 mL). The organic layers were combined to afford 9.5 mL of TfN_3_ solution. Then, to a solution of *para*-bromoaniline (700mg, 4.06mmol) and CuSO_4_ (5 mg) in H_2_O (9.5 mL) was added the TfN_3_ solution, MeOH (31 mL) and Et_3_N (1.6 mL). The reaction mixture was stirred overnight at room temperature. Then solid NaHCO_3_ (1.0 g) was added carefully and the organic solvents were evaporated. The aqueous residue was extracted with EtOAc (3 × 100 mL), and the organic layers were combined, dried (Na_2_SO_4_), and concentrated *in vacuo* to give a yellow liquid. Purification by column chromatography (EtOAc/heptane, 1/20) afforded 1-azido-4-bromobenzene (630mg, 78%) as a colorless liquid. *R*_F _0.61 (EtOAc/heptane, 1/8); ^1^H-NMR (CDCl_3_, 400 MHz) δ 7.44 (d, *J* = 8.9 Hz, 2H), 6.89 (d, *J* = 8.9 Hz, 2H); ^13^C-NMR (CDCl_3_, 75 MHz) δ 138.7, 132.3, 120.1, 117.2.

#### 1-(Azidomethyl)-4-methoxybenzene

To a solution of 1-(chloromethyl)-4-methoxybenzene (400 μL, 2.9 mmol) in 2 mL of DMF was added NaN_3_ (188 mg, 2.9 mmol). The reaction mixture was stirred at 60 °C for 5 h. The reaction was then quenched with H_2_O and extracted with Et_2_O. The organic layers were washed with H_2_O and brine, dried (Na_2_SO_4_) and concentrated in *vacuo*. Purification by column chromatography (EtOAc/heptane, 1/20) gave 1-(azidomethyl)-4-methoxybenzene (410 mg, 87%) as a colorless liquid. *R*_F _0.36 (EtOAc/heptane, 1/8); ^1^H-NMR (CDCl_3_, 400 MHz) δ 7.23(d, *J* = 8.8 Hz, 2H), 6.90 (d, *J* = 8.8 Hz, 2H), 4.24 (s, 2H), 3.78 (s, 3H); ^13^C-NMR (CDCl_3_, 75 MHz) δ 159.2, 129.2, 126.9, 113.7, 54.7, 53.9.

#### 1-Azido-4-methoxybenzene

To a solution of NaN_3_ (1.5 g, 24.3mmol) in a mixture of H_2_O/CH_2_Cl_2_ (7.5 mL, 1:1 v/v) at 0 °C, was added Tf_2_O (2.01 mL, 12.15mmol). The reaction mixture was stirred at room temperature for 2 h. After quenching with aqueous NaHCO_3_, the layers were separated and the aqueous layer was extracted with CH_2_Cl_2_ (5.75 mL). The organic layers were combined to afford 9.5 mL of TfN_3_ solution. Then, to a solution of *para*-methoxyaniline (500mg, 4.06mmol) and CuSO_4_ (5 mg) in H_2_O (9.5 mL) was added the TfN_3_ solution, MeOH (31 mL) and Et_3_N (1.6 mL). The reaction mixture was stirred overnight at room temperature. Then solid NaHCO_3_ (1.0 g) was added carefully and the organic solvents were evaporated. The aqueous residue was extracted with EtOAc (3 × 100 mL), and the organic layers were combined, dried (Na_2_SO_4_), and concentrated *in vacuo* to give yellow liqued. Purification by column chromatography (EtOAc/heptane, 1/20) afforded 1-azido-4-methoxybenzene (490mg, 81%) as a colorless liquid. *R*_F _0.42 (EtOAc/heptane, 1/8); ^1^H-NMR (CDCl_3_, 400 MHz) δ 6.94 (m, 2H), 6.87 (m, 2H), 3.78 (s, 3H); ^13^C-NMR (CDCl_3_, 75 MHz) δ156.5, 131.8, 119.5, 114.6, 55.0.

#### 1-(Azidomethyl)-2-methoxybenzene

To a solution of 1-(chloromethyl)-2-methoxybenzene (400 μL, 2.9 mmol) in 2 mL of DMF was added NaN_3_ (188 mg, 2.9 mmol). The reaction mixture was stirred at 60 °C for 5 h. The reaction was then quenched with H_2_O and extracted with Et_2_O. The organic layers were washed with H_2_O and brine, dried (Na_2_SO_4_) and concentrated in *vacuo*. Purification by column chromatography (EtOAc/heptane, 1/20) gave 1-(azidomethyl)-2-methoxybenzene (406 mg, 86%) as a colorless liquid. *R*_F _0.44 (EtOAc/heptane, 1/8). ^1^H-NMR (CDCl_3_, 400 MHz) δ 7.29(m, 2H), 6.94 (m, 2H), 4.35 (s, 2H), 3.84 (s, 3H).^13^C-NMR (CDCl_3_, 75 MHz) δ 157.2, 129.6, 129.3, 123.4, 120.1, 110.1, 54.8, 49.7.

### General Procedure for Copper (I)-Catalyzed Azido-Alkyne Cycloaddition (CuAAC) (Compounds ***9aA-10g***), exemplified for compound ***9aA***

To a solution of **9a** (15 mg, 0.020 mmol) in a mixture of MeCN/H_2_O (2 mL, 20:1 v/v), was added linker **A** (1.8 mg, .010 mmol). The reactionmixture was stirred at room temperature in the presence of a Cu-wired stirringbar. After 3 h, the Cu-wire was removed and stirring wasprolonged until all starting material was consumed. The volatiles were removed in *vacuo.* Purification by column chromatography (MeOH/CH_2_Cl_2_, 1/9) afforded **9aA** (11 mg, 63%) as a white solid. HRMS (ESI) *m/z* calcd. for C_52_H_80_N_18_O_8_ (M+H)^+^: 1085.6484, found: 1085.6464; RP-HPLC: *t*_R_ = 15.42 min.

*Dimer*** 9aB:** Yield 57%, white solid. HRMS (ESI) *m/z* calcd. for C_52_H_80_N_18_O_8_ (M+H)^+^: 1085.6484, found: 1085.6482; RP-HPLC: *t*_R_ = 17.23 min.

*Dimer* **9aC:** Yield 61%, white solid.^ 1^H-NMR (MeOD, 400 MHz): δ 7.95 (s, 2H), 7.78 (d, *J* = 6.4 Hz, 2H), 6.91 (s, 2H), 6.81 (dd, *J* = 6.6, 1.4 Hz, 2H), 5.09 (d, *J* = 7.7 Hz, 2H), 4.36 (t, *J* = 7.0 Hz, 4H), 4.10 (m, 2H), 3.89 (s, 2H), 3.77 (t, *J* = 6.0 Hz, 4H), 3.65 (t, *J* = 9.2 Hz, 2H), 3.48 (m, 6H), 3.35 (m, 4H), 3.21 (m, 6H), 2.39 (s, 6H), 2.36 (dt, *J* = 8.2, 3.8 Hz, 2H), 1.91 (m, 4H), 1.72 (q, *J* = 12.4 Hz, 2H), 1.27 (m, 2H). HRMS (ESI) *m/z* calcd for C_49_H_82_N_18_O_8_ (M+H)^+^: 1051.6641, found: 1051.6577; RP-HPLC: *t*_R_ = 16.33 min.

*Dimer* **9aD:** Yield 60%, white solid. HRMS (ESI) *m/z* calcd. for C_48_H_80_N_18_O_8_ (M+H)^+^: 1037.6484, found: 1037.6448; RP-HPLC: *t*_R_ = 16.28 min.

*Dimer*** 9aE:** Yield 87%, white solid. HRMS (ESI) *m/z* calcd. for C_29_H_43_N_9_O_4_ (M+H)^+^: 582.3516, found: 582.3516; RP-HPLC: *t*_R_ = 15.12 min.

*Dimer*** 9bA:** Yield 73%, white solid. HRMS (ESI) *m/z* calcd. for C_52_H_80_N_18_O_8_ (M+H)^+^: 1085.6412, found: 1085.6419; RP-HPLC: *t*_R_ = 16.72 min.

*Dimer***9bB:** Yield 65%, white solid. HRMS (ESI) *m/z* calcd. for C_52_H_80_N_18_O_8_ (M+H)^+^: 1085.6484, found: 1085.6442; RP-HPLC: *t*_R_ = 16.44 min.

*Dimer***9bC:** Yield 66%, white solid. HRMS (ESI) *m/z* calcd. for C_49_H_82_N_18_O_8_ (M+H)^+^: 1051.6641, found: 1051.6641; RP-HPLC: *t*_R_ = 17.11 min.

*Dimer*** 9bD:** Yield 65%, white solid. HRMS (ESI) *m/z* calcd. for C_48_H_80_N_18_O_8_ (M+H)^+^: 1037.6484, found: 1037.6460; RP-HPLC: *t*_R_ = 16.81 min.

*Dimer*** 9bE:** Yield 92%, white solid. HRMS (ESI) *m/z* calcd. for C_29_H_43_N_9_O_4_ (M+H)^+^: 582.3516, found: 582.3516; RP-HPLC: *t*_R_ = 16.19 min.

*Dimer* **9cA:** Yield 60%, white solid. HRMS (ESI) *m/z* calcd. for C_60_H_84_N_18_O_8_ (M+H)^+^: 1185.6797, found: 1185.6878; RP-HPLC: *t*_R_ = 22.72 min.

*Dimer* **9cB:** Yield 58%, white solid; HRMS (ESI) *m/z* calcd. for C_60_H_84_N_18_O_8_ (M+H)^+^: 1185.6797, found: 1185.6781; RP-HPLC: *t*_R_ = 24.13 min.

*Dimer*** 9cC:** Yield 69%, white solid.^ 1^H-NMR (MeOD, 400 MHz): δ 8.09 (s, 2H), 7.96 (d, *J* = 8.1 Hz, 2H), 7.82 (s, 2H), 7.71 (t, *J* = 7.2 Hz, 2H), 7.49 (t, *J* = 8.1 Hz, 2H), 6.99 (s, 2H), 5.24 (d, *J* = 7.4 Hz, 2H), 4.44 (s, 2H), 4.34 (t, *J* = 7.0 Hz, 4H), 3.95 (s, 4H), 3.65 (t, *J* = 9.1 Hz, 4H), 3.38–3.60 (m, 8H), 3.27 (t, *J* = 6.6 Hz, 4H), 3.16 (m, 4H), 2.95 (s, 2H), 2.63 (s, 4H), 2.32 (dt, *J* = 12.2, 4.1 Hz, 2H), 1.87 (m, 4H), 1.69 (q, *J* = 12.2 Hz, 2H), 1.26 (m, 2H); HRMS (ESI) *m/z* calcd. for C_57_H_86_N_18_O_8_ (M+H)^+^: 1151.6954, found: 1151.6945; RP-HPLC: *t*_R_ = 21.61 min.

*Dimer* **9cD:** Yield 66%, white solid; HRMS (ESI) *m/z* calcd. for C_56_H_84_N_18_O_8_ (M+H)^+^: 1137.6797, found: 1137.6793; RP-HPLC: *t*_R_ = 23.12 min.

*Dimer*** 9cE:** Yield 90%, white solid; HRMS (ESI) *m/z* calcd. for C_33_H_45_N_9_O_4_ (M+H)^+^: 632.3672, found: 632.3669; RP-HPLC: *t*_R_ = 19.87 min.

*Compound*** 10a:** Yield 79%, white solid;^ 1^H-NMR (MeOD, 400 MHz): δ 8.04 (s, 1H), 8.02 (d, J = 8.4 Hz, 1H), 7.77 (t, *J* = 7.7 Hz, 1H), 7.55 (t, *J* = 7.7 Hz, 1H), 7.50 (d, *J* = 8.2 Hz, 2H), 7.24 (d, *J* = 8.2 Hz, 2H), 5.55 (s, 2H), 5.16 (d, *J* = 7.2 Hz, 1H), 4.17 (s, 3H), 3.98 (t, *J* = 6.0 Hz, 2H), 3.65 (t, *J* = 9.0 Hz, 1H), 3.56 (m, 1H), 3.48 (t, *J* = 9.7 Hz, 4H), 3.37 (dd, *J* = 12.8, 2.3 Hz, 1H), 3.20 (m, 4H), 2.69 (s, 3H), 2.60 (m, 2H), 2.36 (dt, *J* = 8.1, 3.7 Hz, 1H), 1.71 (q, *J* = 12.4 Hz, 1H); HRMS (ESI) *m/z* calcd. for C_33_H_44_N_9_O_4_Br (M+H)^+^: 710.2777, found: 710.2761; RP-HPLC: *t*_R_ = 20.43 min.

*Compound*** 10b:** Yield 71%, white solid; HRMS (ESI) *m/z* calcd. for C_32_H_42_N_9_O_4_Br (M+H)^+^: 696.2621, found: 696.2664; RP-HPLC: *t*_R_ = 21.18 min.

*Compound*** 10c:** Yield 77%, white solid; ^1^H-NMR (MeOD, 400 MHz): δ 8.03 (s, 1H), 8.01 (d, *J* = 8.1 Hz, 1H), 7.76 (t, *J* = 7.7 Hz, 1H), 7.54 (t, *J* = 7.7 Hz, 1H), 7.28 (d, *J* = 8.7 Hz, 2H), 6.88 (d, *J* = 8.7 Hz, 2H), 5.49 (s, 2H), 5.23 (d, *J* = 7.0 Hz, 1H), 4.36 (s, 2H), 4.28 (t, *J* = 9.5 Hz, 1H), 3.99 (t, *J* = 5.5 Hz, 2H), 3.74 (s, 3H), 3.66 (t, *J* = 9.1 Hz, 1H), 3.63 (d, *J* = 11.5 Hz, 1H), 3.57 (dd, *J* = 12.8, 6.4 Hz, 1H), 3.36–3.55 (m, 4H), 3.31 (s, 3H), 2.84 (m, 2H), 2.68 (s, 2H), 2.37 (dt, *J* = 12.0, 4.0 Hz, 1H), 1.73 (q, *J* = 12.5 Hz, 1H); HRMS (ESI) *m/z* calcd. for C_34_H_47_N_9_O_5_ (M+H)^+^: 662.3778, found: 662.3758; RP-HPLC: *t*_R_ = 21.34 min.

*Compound*** 10d:** Yield 72%, white solid; HRMS (ESI) *m/z* calcd. for C_33_H_45_N9O_5_ (M+H)^+^: 648.3621, found: 648.3600; RP-HPLC: *t*_R_ = 20.63 min.

*Compound*** 10e:** Yield 81%, white solid;^ 1^H-NMR (MeOD, 400 MHz): δ 8.02 (d, *J* = 8.2 Hz, 1H), 7.97 (s, 1H), 7.77 (dd, *J* = 8.2, 7.2 Hz, 1H), 7.55 (dd, *J* = 8.2, 7.2 Hz, 1H), 7.33 (m, 1H), 7.23 (dd, *J* = 7.4, 1.6 Hz, 1H), 7.00 (d, *J* = 8.3 Hz, 1H), 6.92 (dt, *J* = 7.5, 1.0 Hz, 1H), 5.55 (s, 2H), 5.19 (d, *J* = 8.3 Hz, 1H), 4.24 (s, 3H), 3.99 (t, *J* = 5.9 Hz, 2H), 3.83 (s, 3H), 3.68 (t, *J* = 9.2 Hz, 1H), 3.35–3.60 (m, 5H), 2.84 (m, 3H), 2.69 (s, 4H), 2.36 (dt, *J* = 12.1, 4.0 Hz, 1H), 1.71 (q, *J* =12.6 Hz, 1H); HRMS (ESI) *m/z* calcd for C_34_H_47_N_9_O_5_ (M+H)^+^: 662.3778, found: 662.3769; RP-HPLC: *t*_R_ = 21.15 min.

*Compound*** 10f:** Yield 70%, white solid; HRMS (ESI) *m/z* calcd. for C_34_H_48_N_10_O_4_ (M+H)^+^: 661.3938, found: 661.3914; RP-HPLC: *t*_R_ = 21.32 min.

*Compound***10g:** Yield 68%, white solid; HRMS (ESI) *m/z* calcd. for C_31_H_50_N_10_O_4_ (M+H)^+^: 627.4094, found: 627.4084; RP-HPLC: *t*_R_ = 21.12 min.

### FRET-based oxygen-sensitive assay *[71]*

A typical experiment using a 96-well U-bottom plate was performed as follows. Serial dilutions of a freshly grown cell culture were made in LB to achieve 10^9^ to 10^2^ colony-forming units (cfu, confirmed by colony plate counting). Wells were prepared in triplicate by filling each well with 180 μl of the cell dilution plus 20 μL of Cy3-labeled hemocyanin solution (0.5–1 μM end-concentration). To avoid evaporation and rapid oxygen diffusion, 50 μL of mineral oil was added on top of each well. For antibiotic resistance tests, the initial number of cells was 10^7^ cfu/well. Kanamycin was added to a final concentration of 0.05 to 5000 μg/mL. Controls were performed by adding Cy3 or Cy3-labeled BSA instead of Cy3-labeled hemocyanin to the cell culture as well as by using unlabeled hemocyanin. The fluorescence of Cy3-labeled hemocyanin in LB without cells was also monitored as a control in the absence of oxygen consumption.

## Conclusions

A set of dimers of aminoglycoside analogues has been synthesized from natural neomycin via a straightforward route. Cleavage of both diaminoglucose pyranoses was followed by installation of an N-propargylated morpholine. Subsequent selective oxidation of the primary alcohol and immediate conjugation to arylamine by reductive amination led to three different 2-deoxystreptamine conjugates. Dimerization of the resulting conjugates with a range of bisazides afforded a small library of potential RNA-binding ligands in a straightforward synthetic route involving minimal protecting group transformations. All compounds were evaluated for antibacterial activity employing a novel fluorescence-based cell viability essay and compared to kanamycin. Several of the compounds, dimers and monomers, showed antibacterial activity, with values lying in the high micromolar range. Further optimization of the conjugates is therefore required, e.g., by introduction of longer spacers between the arylamines and the morpholine to allow cooperative binding. Similar reasoning may be applied to the dimers, as well as to the monofunctionalized morpholines.
